# Dysregulated methylation at imprinted genes in prostate tumor tissue detected by methylation microarray

**DOI:** 10.1186/1471-2490-13-37

**Published:** 2013-07-26

**Authors:** Daniel I Jacobs, Yingying Mao, Alan Fu, William Kevin Kelly, Yong Zhu

**Affiliations:** 1 Yale School of Public Health, Yale University School of Medicine, New Haven, CT, USA; 2 Department of Epidemiology and Health Statistics, Zhejiang University School of Public Health, Hangzhou, Zhejiang Province, China; 3 Department of Medical Oncology and Urology, Thomas Jefferson University, Philadelphia, Pennsylvania, USA

**Keywords:** Imprinting, Prostate cancer, Differential methylation, *DLK1*, *PLAGL1*, *SLC22A18*, *TP73*, *WT1*

## Abstract

**Background:**

Imprinting is an important epigenetic regulator of gene expression that is often disrupted in cancer. While loss of imprinting (LOI) has been reported for two genes in prostate cancer (*IGF2* and *TFPI2*), disease-related changes in methylation across all imprinted gene regions has not been investigated.

**Methods:**

Using an Illumina Infinium Methylation Assay, we analyzed methylation of 396 CpG sites in the promoter regions of 56 genes in a pooled sample of 12 pairs of prostate tumor and adjacent normal tissue. Selected LOI identified from the array was validated using the Sequenom EpiTYPER assay for individual samples and further confirmed by expression data from publicly available datasets.

**Results:**

Methylation significantly increased in 52 sites and significantly decreased in 17 sites across 28 unique genes (*P* < 0.05), and the strongest evidence for loss of imprinting was demonstrated in tumor suppressor genes *DLK1*, *PLAGL1*, *SLC22A18*, *TP73*, and *WT1*. Differential expression of these five genes in prostate tumor versus normal tissue using array data from a publicly available database were consistent with the observed LOI patterns, and *WT1* hypermethylation was confirmed using quantitative DNA methylation analysis.

**Conclusions:**

Together, these findings suggest a more widespread dysregulation of genetic imprinting in prostate cancer than previously reported and warrant further investigation.

## Background

Genomic imprinting is the epigenetic phenomenon by which alleles of select genes are differentially expressed according to the parent of origin [1]. In humans, approximately 65 genes have been validated as imprinted [2]. It has been suggested that imprinting is regulated primarily by DNA methylation of imprinting control regions (ICRs), which is established in the germ line and maintained throughout subsequent development [3].

Loss of monoallelic expression at imprinted genes, known as loss of imprinting (LOI), has been associated with many cancer types including leukemia, colorectal, liver, and lung cancer [4] and may play a role as an early driver in tumorigenesis [5]. Abnormal methylation of imprinted genes can be detrimental given frequent roles in promoting and restricting cellular growth. For example, loss of methylation of the maternal allele of insulin-like growth factor-II (*IGF2*) has been associated with increased expression of the growth-promoting gene in Wilms’ tumor [6].

While studies have suggested a role for *IGF2* and tissue factor pathway inhibitor-2 (*TFPI2*) LOI in prostate cancer [7-9], the literature is restricted largely to these two genes. Here, we present a comprehensive investigation of methylation patterns at imprinted genes in prostate cancer. Our results indicate an overall dysregulation of imprinted gene methylation levels in prostate tumor tissue as compared to adjacent normal tissue, with pronounced gain of methylation at five tumor suppressor genes.

## Methods

### Study subjects

Procedures for participant recruitment have been described previously [10]. Briefly, study subjects were identified via the Yale Cancer Center Rapid Case Ascertainment system and all patients consented to the donation of tissue to the Yale-New Haven Hospital (YNHH) tissue bank. Samples were obtained according to protocols approved by the Research Ethics Board from YNHH, New Haven County, Connecticut and the Connecticut Department of Public Health Human Investigations Committee. Tissue sections from seventeen pairs of formalin-fixed paraffin-embedded (FFPE) prostate cancers and corresponding adjacent normal tissue specimens, obtained from patients who had undergone surgery between 2005 and 2009 at YNHH, were mounted on slides and examined by an expert pathologist. Gleason grades varied between specimens, with a composite score ranging from 6 to 9. No subjects who had received either chemo- or radio-therapy were included in the study.

### Isolation of genomic DNA

Sections of tumor and adjacent normal tissue were pathologically reviewed, manually microdissected, and collected into 1.5 ml microtubes. The DNeasy Blood & Tissue Kit (Qiagen, Valencia, CA) was used to isolate genomic DNA according to the manufacturer’s protocols.

### Methylation assay

Equal amounts of DNA from tumor and matching adjacent normal tissue from twelve subjects (a sufficient amount of DNA was unavailable for five of the seventeen subjects) were combined by tissue type for CpG methylation microarray analysis. Methylation of imprinted genes was assessed using the Illumina Infinium HumanMethylation27 Array (Illumina, Inc., San Diego, CA). The CpG sites of the imprinted genes were located within promoter regions, ranging from 3 to 1,495 bp upstream of the transcription start site (average distance: 426 ± 373 bp). A methylation index (β) was obtained for each site, which is a continuous variable ranging between 0 and 1 representing the ratio of the intensity of the methylated-probe signal to the total locus signal intensity (a β value of 0 corresponds to no methylation while a value of 1 corresponds to 100% methylation at the specific CpG locus measured). Complete array data have been uploaded to the Gene Expression Omnibus (GEO) database (http://www.ncbi.nlm.nih.gov/geo/; accession number GSE26319).

### Validation by quantitative DNA methylation analysis

In order to confirm methylation microarray results we carried out quantitative DNA methylation analysis using Sequenom’s EpiTYPER assay (Sequenom, Inc., San Diego, CA). Methylation levels at *WT1* were analyzed using tumor and adjacent normal tissue DNA from five subjects (non-pooled) for which DNA was of sufficient concentration. Analysis was conducted using pre-validated primers from the Sequenom’s Imprinting EpiPanel designed to target imprinting control regions of known imprinted genes (Amplicon WT1.ALT.TRANSCRIPT_05; Forward primer: GTAGGGGTTAGGGGAGGTAAAGT; Reverse primer: CCCAATCACAATACAACTACAATCA). Average methylation levels in tumor versus normal tissue were compared for individual CpG sites and for overall *WT1* methylation.

### Expression confirmation in publicly available datasets

We used the Oncomine expression profiling database version 4.4 (http://www.oncomine.org; accessed March 3rd, 2012) to search for expression array comparisons between prostate cancer tissue and normal tissue (either from adjacent normal tissue or healthy controls) in five genes with the strongest evidence of LOI in our dataset. We searched the database for expression differences in human prostate cancer using the gene symbol as the keyword (e.g. “DLK1”).

### Statistical analysis

The Infinium methylation data were analyzed using Illumina's GenomeStudio software, which employs a custom model to yield a *DiffScore* and p-value for each CpG site based on a comparison of the mean methylation level in tumor tissue versus that of adjacent normal tissue. To control for multiple comparisons, adjustments were made in order to obtain an adjusted *P* value (designed as the false discovery rate, or *Q* value) for each observation using the method originally proposed by Benjamini-Hochberg [11]. CpG sites were defined as differentially methylated if the *Q* values obtained were < 0.05. Average methylation levels derived from SEQUENOM EpiTYPER analysis were compared between tumor and adjacent normal samples using a two sample t-test.

## Results

### Global disruption of methylation at imprinted genes

**Figure 1:**
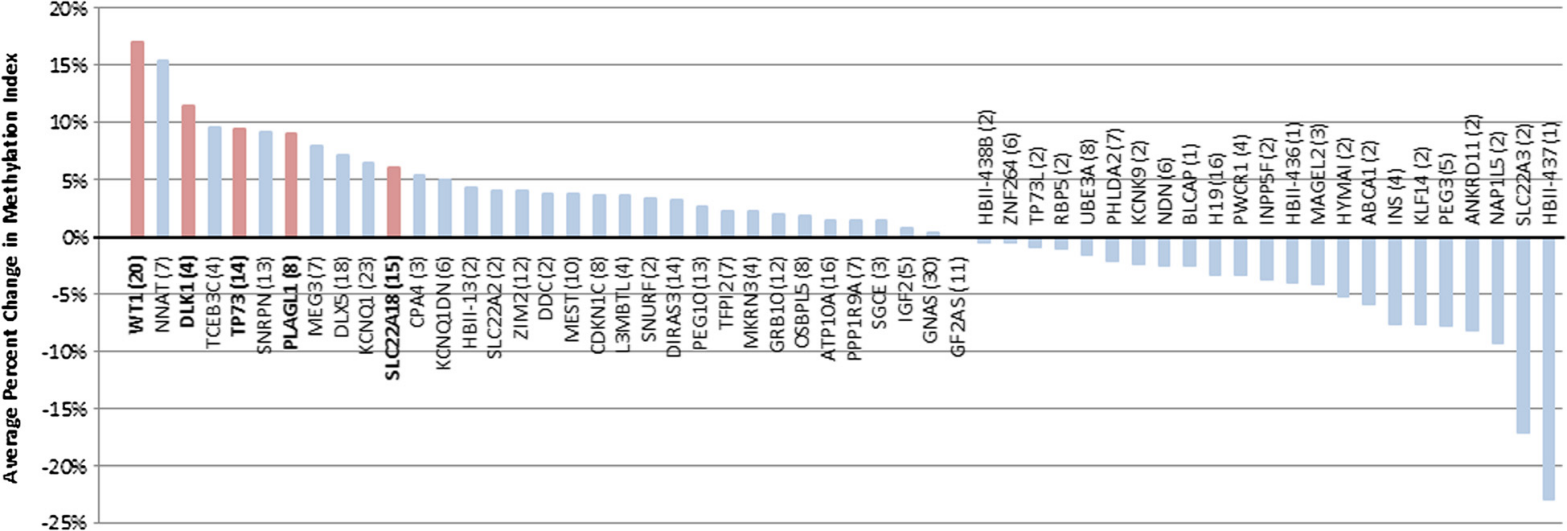
**Average percent change in methylation index (β) for tumor tissue (relative to normal) across all measured CpG sites for all imprinted genes.** Numbers in parentheses indicate the number of CpG sites measured, and red bars indicate genes selected for further analysis. Of the 56 genes, 13 demonstrated higher average methylation indices of at least 5%, and 9 demonstrated lower average methylation indices of at least 5%.

Based on our analysis of a pooled sample of 12 pairs of prostate tumor and adjacent normal tissues, our results demonstrate an overall disruption of methylation patterns of imprinted genes in tumor tissue. Among 397 CpG sites analyzed in 56 imprinted genes that were covered by the Illumina Infinium HumanMethylation27 array, the methylation index (β) in adjacent normal tissues was 0.453 on average, and in prostate cancer tissue methylation was significantly higher at 52 sites (13.1%) and significantly lower at 17 (4.3%) sites (*P* < 0.05), jointly spanning 28 unique genes (Table 1). Average percent changes in the methylation index were calculated for each of the 56 imprinted genes, in which percent changes were averaged across the set of corresponding CpG sites for each gene (mean number of CpG sites/gene: 7.1 ± 6.2). Of the 56 genes, 13 demonstrated a higher average methylation of at least 5%, and 9 demonstrated a lower average methylation of at least 5% (Figure 1).

**Table 1:** **Methylation indices** (**β**) **for CpG sites with significant methylation changes** (***P*** < **0**.**05**)

**Gene**	**CpG site**	**Normal tissue(β1)**	**Tumor tissue(β2)**	**Change in methylation index(β2-β1)**	**Fold change**	**Gene**	**CpG site**	**Normal tissue(β1)**	**Tumor tissue(β2)**	**Change in methylation index(β2-β1)**	**Fold change**
ATP10A	cg10734665	0.717	0.394	−0.323	−1.820	IGF2	cg20339650	0.060	0.254	0.194	4.233
ATP10A	cg11015241	0.453	0.714	0.261	1.576	IGF2AS	cg13791131	0.050	0.253	0.203	5.060
CDKN1C	cg05559445	0.360	0.567	0.207	1.575	INS	cg25336198	0.732	0.530	−0.202	−1.381
DIRAS3	cg09118625	0.657	0.847	0.190	1.289	KCNQ1	cg06719391	0.329	0.092	−0.237	−3.576
DLK1	cg17412258	0.088	0.380	0.291	4.318	KCNQ1	cg20751395	0.847	0.690	−0.157	−1.228
DLX5	cg11500797	0.451	0.652	0.201	1.446	KCNQ1	cg17820828	0.087	0.263	0.176	3.023
DLX5	cg24115040	0.368	0.581	0.212	1.579	KCNQ1	cg12578166	0.552	0.813	0.261	1.473
DLX5	cg20080624	0.549	0.767	0.219	1.397	KCNQ1	cg01734338	0.530	0.900	0.369	1.698
DLX5	cg06537230	0.057	0.380	0.323	6.667	KCNQ1DN	cg05656180	0.507	0.707	0.200	1.394
DLX5	cg06911084	0.320	0.709	0.389	2.216	KCNQ1DN	cg13081704	0.557	0.759	0.202	1.363
GNAS	cg01355739	0.882	0.640	−0.243	−1.378	KLF14	cg06533629	0.548	0.319	−0.229	−1.718
GNAS	cg17414107	0.360	0.125	−0.235	−2.880	MEG3	cg25836301	0.558	0.745	0.187	1.335
GNAS	cg06044900	0.284	0.123	−0.161	−2.309	MEG3	cg09280976	0.643	0.870	0.226	1.353
GNAS	cg07284407	0.485	0.682	0.197	1.406	MEST	cg09872616	0.138	0.309	0.171	2.239
GNAS	cg01565918	0.457	0.720	0.263	1.575	MEST	cg17347253	0.338	0.585	0.247	1.731
GNAS	cg09437522	0.299	0.565	0.266	1.890	NAP1L5	cg12759554	0.714	0.407	−0.307	−1.754
H19	cg25852472	0.801	0.513	−0.288	−1.561	NNAT	cg23566503	0.645	0.823	0.178	1.276
H19	cg10602543	0.790	0.560	−0.229	−1.411	NNAT	cg12862537	0.712	0.903	0.191	1.268
H19	cg11492040	0.626	0.414	−0.212	−1.512	NNAT	cg18433380	0.401	0.625	0.224	1.559
H19	cg23977670	0.888	0.721	−0.167	−1.232	NNAT	cg10642330	0.567	0.865	0.299	1.526
HBII-437	cg08993557	0.686	0.458	−0.228	−1.498	PEG10	cg25524350	0.141	0.581	0.440	4.121
PLAGL1	cg08263357	0.429	0.678	0.249	1.580	TP73	cg25115460	0.378	0.745	0.367	1.971
PLAGL1	cg17895149	0.128	0.418	0.290	3.266	TP73	cg16607065	0.311	0.708	0.397	2.277
PLAGL1	cg25350411	0.298	0.611	0.313	2.050	WT1	cg04096767	0.463	0.670	0.207	1.447
PPP1R9A	cg11164400	0.435	0.725	0.291	1.667	WT1	cg22511262	0.265	0.500	0.235	1.887
SLC22A18	cg18655584	0.677	0.860	0.182	1.270	WT1	cg15446391	0.322	0.571	0.249	1.773
SLC22A18	cg03336167	0.234	0.878	0.643	3.752	WT1	cg13301003	0.320	0.580	0.260	1.813
SLC22A3	cg25313204	0.956	0.626	−0.330	−1.527	WT1	cg01693350	0.505	0.781	0.276	1.547
SNRPN	cg24993443	0.549	0.793	0.244	1.444	WT1	cg05222924	0.336	0.617	0.281	1.836
SNRPN	cg11265941	0.297	0.595	0.298	2.003	WT1	cg12006284	0.315	0.724	0.410	2.298
SNRPN	cg22678136	0.422	0.827	0.405	1.960	WT1	cg22533573	0.075	0.575	0.500	7.667
TCEB3C	cg02432101	0.644	0.861	0.218	1.337	ZIM2	cg06244906	0.796	0.553	−0.243	−1.439
TP73	cg00565688	0.580	0.230	−0.351	−2.522	ZIM2	cg02162069	0.561	0.751	0.190	1.339
TP73	cg26208930	0.601	0.866	0.265	1.441	ZIM2	cg16519742	0.635	0.867	0.232	1.365
TP73	cg03846767	0.430	0.749	0.319	1.742						

### Strongest evidence for methylation dysregulation at five imprinted genes

**Figure 2:**
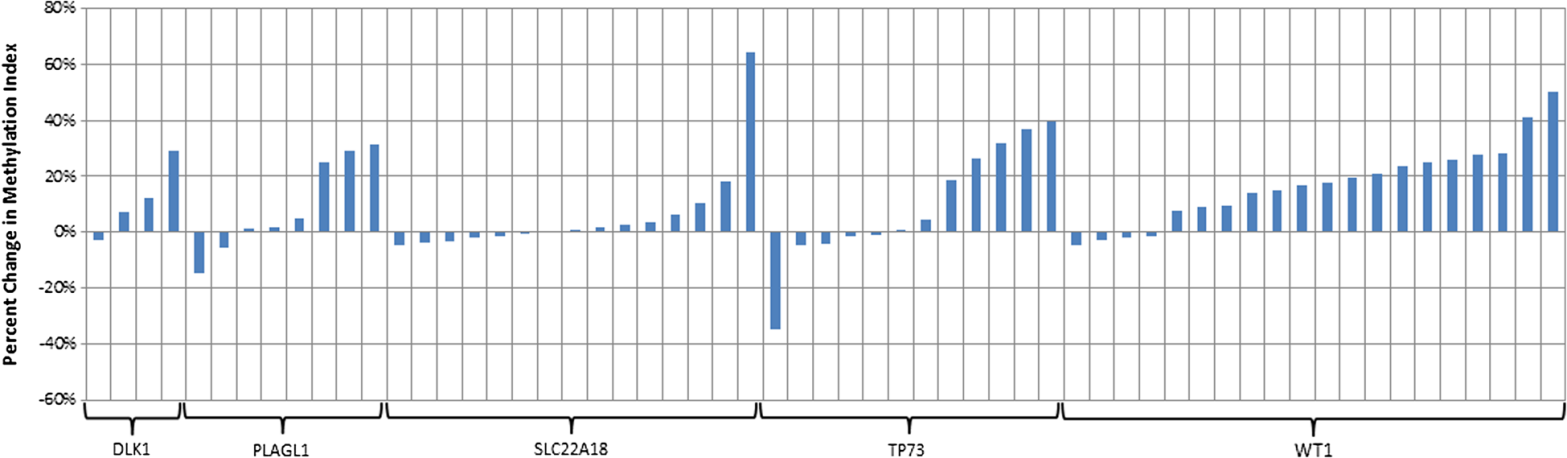
**Changes in methylation in tumor tissue by CpG Site in *DLK1*, *PLAGL1*, *SLC22A18*, *TP73*, and *WT1*.** Bars represent the percentage change in the methylation index (β) in pooled tumor samples relative to pooled adjacent tissue samples. All measured CpG sites for the five genes are presented.

Results from five genes were particularly notable based on the magnitude of methylation change from adjacent normal tissue and the consistency in the direction of these changes across multiple CpG sites (Figure 2). CpG site cg17412258, 212 bp upstream of the *DLK1* transcription start site, showed 4.32 fold higher methylation in the tumor samples relative to the adjacent normal tissue samples, with a 5% higher average methylation index for the other CpG sites. *SLC22A18* demonstrated similar methylation changes, where 3.75 fold higher methylation was observed at CpG site cg03336167 with a tendency toward higher methylation at remaining sites. Three CpG sites in the promoter region of *PLAGL1* showed statistically significant higher methylation (*P* < 0.05), including 3.27 fold higher methylation at cg17895149. Four CpG sites in the proximal *TP73* promoter region demonstrated statistically significant higher methylation indices (1.44, 1.74, 1.97, and 2.28 fold higher methylation) (*P* < 0.05), while one site further upstream from the transcription start site (917 bp) showed statistically significantly lower methylation (*P* < 0.05). *WT1* demonstrated the greatest consistency in methylation aberration, with increased methylation at 16 of 20 CpG sites. This increased methylation was statistically significant (*P* < 0.05) at eight CpG sites including 7.67 fold higher methylation at cg22533573. All five of the identified genes (*DLK1*, *PLAGL1*, *SLC22A18*, *TP73*, and *WT1*) have presumed tumor suppressing functions and taken together demonstrate a strong tendency towards higher methylation at the CpG sites assessed.

### Validation of WT1 hypermethylation by quantitative DNA methylation analysis

We sought to confirm the hypermethylation observed at *WT1* using SEQUENOM’s EpiTYPER quantitative methylation assay. Results were consistent with the prior analysis by microarray, demonstrating roughly 2–4 fold hypermethylation across all eight CpG sites analyzed in the *WT1* imprinting control region (including cg22533573, as discussed above) in comparing individual tumor and adjacent normal tissue pairs. Averaged across the eight analyzed CpG sites and five tumor-normal tissue pairs with adequate DNA for this confirmation, we observed a 2.04-fold increase in methylation in tumor tissue relative to adjacent normal tissue (average normal methylation level: 25.2%; average tumor methylation level: 51.4%; *P* = 0.0104).

### Expression confirmation of differentially methylated genes using public data

**Figure 3:**
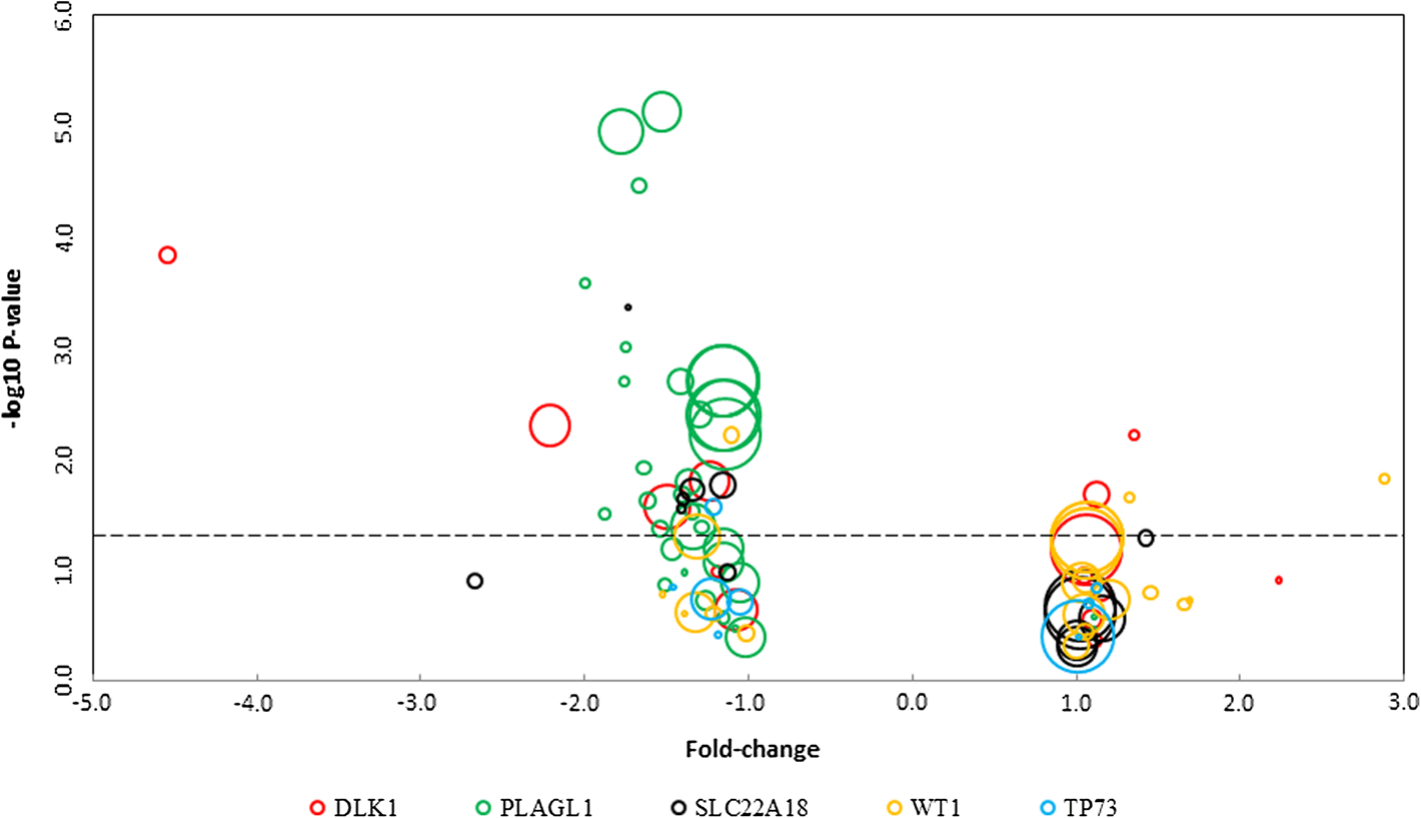
**Expression of five selected genes (*DLK1*, *PLAGL1*, *SLC22A18*, *TP73*, and *WT1*) in prostate cancer tissue relative to normal tissue in all studies of prostate cancer identified from the publicly available Oncomine expression database.** Study results are plotted according to the -log(P-value) and expression fold-change in tumor versus normal tissue, and sample size is represented by the size of the circles (range: n = 12 to n = 160). The dotted line represents statistical significance at *P* = 0.05.

We used the Oncomine expression profiling database to search for expression array comparisons between prostate cancer tissue and prostate tissue from healthy controls in *DLK1*, *PLAGL1*, *SLC22A18*, *TP73*, and *WT1*; expression data for all available studies are presented in Figure 3. Six of fourteen studies reported statistically significant (*P* < 0.05) expression changes for *DLK1*, demonstrating lower expression of the gene in prostate cancer tissues relative to healthy control tissues in four studies and higher expression in two. These studies reported expression fold changes of −4.547 (n = 34) [12], -2.216 (n = 89) [13], -1.496 (n = 102) [14], -1.242 (n = 88) [15], 1.124 (n = 57) [16], and 1.350 (n = 21) [17], respectively. Of 38 studies, 27 reported significantly lower expression of *PLAGL1* in prostate cancer tissue relative to healthy controls. These studies reported lower expression fold changes ranging from 1.143 [18] to 1.995 [17], and no study reported higher expression of the gene in tumor tissue. Five of thirteen studies were identified that showed significantly lower expression for *SLC22A18*, with fold changes of −1.734 (n = 13) [19], -1.411 (n = 21) [17], -1.400 (n = 26) [20], -1.341 (n = 52) [21], and −1.157 (n = 57) [16]. Four out of 22 studies showed significant *WT1* expression changes, including two studies demonstrating lower expression fold changes of −1.315 (n = 102) [14], and −1.106 (n = 35) [22], and two studies showing significantly higher expression with a fold change of 1.327 (n = 21) [17] and 2.878 (n = 21) [17]. Of nine studies, one (n = 35) [22] demonstrated a significant change in expression of *TP73*, with a −1.218 fold change.

## Discussion

This work represents the first comprehensive investigation of methylation changes in prostate cancer. Our results demonstrate an overall disruption of methylation at imprinted genes in prostate cancer tissue with a greater tendency toward hypermethylation than hypomethylation. Based on the magnitude and consistency of hypermethylation across multiple CpG sites, the strongest evidence for disrupted methylation patterns at imprinted genes was demonstrated at five tumor suppressor genes: *DLK1*, *PLAGL1*, *SLC22A18*, *TP73*, and *WT1*. Of the five genes, LOI has been reported for *WT1* in Wilms’ tumor development, [23] and has not been reported for the other four genes in the context of cancer development. Statistically significant hypermethylation across eight CpG sites in the *WT1* imprinting control region was confirmed using quantitative DNA methylation analysis (*P* = 0.0104).

All five of the identified genes are presumed tumor suppressors and have been reported to play roles in cancer development. *DLK1*, which encodes a transmembrane protein and is involved in cell differentiation, has been linked to liver cancer development and progression [24,25]. *PLAGL1* is thought to be a transcriptional regulator and has been associated with pheochromocytoma, a tumor of the adrenal grand [26]. *SLC22A18* is a transporter of organic cations, and has been associated with glioma and breast cancer progression and survival [27,28]. *WT1* plays an important role in normal development of the urogenital system, and is named after its association with Wilms’ tumor development [29]. It has also been associated with breast cancer [30], colorectal cancer [31], and thyroid cancer [32]. *TP73* is an important member of the p53 family of cell cycle regulatory proteins, which is likely disrupted in the majority of cancers [33,34]. Under normal imprinting control, *DLK1*, *PLAGL1*, and *WT1* are paternally expressed, while *SLC22A18* and *TP73* are maternally expressed.

We hypothesized that the increased methylation observed would be associated with lower expression of these five genes, as previous research has established the role that DNA methylation plays in regulating gene expression [35,36]. Methylation impacts gene expression by altering chromatin structure and modifying interactions between proteins and DNA [37]. Specifically, methylated promoter CpG islands attract methyl-CpG binding proteins and transcriptional repressors, thereby interfering with transcription factor binding and reducing transcription of the associated gene [38,39]. Accordingly, we queried the Oncomine database to determine if expression of identified genes significantly differed between prostate cancer and normal prostate tissue in publicly available datasets. Evidence was provided to support a tendency towards reduced expression in prostate tumor tissue at *DLK1*, *PLAGL1*, *SLC22A18*, with less conclusive expression data for *WT1* and *TP73*.

Taken together, our results suggest that observed promoter hypermethylation may be involved in the downregulation of these normally imprinted tumor suppressor genes, which may have important functional consequences for the development and progression of prostate cancer. For example, the transmembrane protein DLK1 has been shown to negatively regulate NOTCH1 [40], which is overexpressed in prostate cancer and is associated with human prostate cancer cell invasion [41]; this suggests that DLK1 downregulation may promote cell invasion via increased NOTCH1 expression. Loss of PLAGL1 expression has been associated with progression from benign to metastatic prostate tumors via the acquisition of androgen-independence, which enables prostate cancers to grow in the absence of androgens [42]. Expression of SLC22A18 (also known as TSSC5) has been observed in adult human prostate tissue and may be involved in growth regulation and small molecule transport, including the export of potentially genotoxic substances [43]; underexpression of this protein may consequently increase the risk of tumor formation. Finally, reduced expression of WT1 and TP73 proteins may have major implications for tumorigenesis: WT1 is a transcription factor that has been shown to regulate growth and induce apoptosis when overexpressed in prostate cancer cells [44], and p73 is a p53-family protein that is key to apoptosis and growth arrest in human prostate cancer cells [45].

Previous studies of loss of imprinting in prostate cancer have focused on *TFPI2* and *IGF2*, with one study reporting lower methylation in the former [8] and three studies providing inconsistent reports of methylation changes in the latter [7,9,46]. No significant changes in methylation were identified in our data at *TFPI2* CpG sites, and *IGF2* methylation was higher at one CpG site and lower at the other four that were assessed. The resulting effects on expression of these two genes were variable across studies identified in the Oncomine expression database.

## Conclusions

This study was limited by the inability to assess expression changes for our samples, as we were unable to extract sufficient RNA from our tissue samples to conduct qPCR analyses. It should also be noted that loss of imprinting has been previously observed in normal tissue from cancer patients [47], which suggests that some differential methylation events at these genes would not be detectable in comparing tumor tissue to adjacent normal tissue. Despite these limitations, this study represents the first comprehensive assessment of methylation changes in prostate cancer. Our results suggest an overall tendency towards disruption of methylation at imprinted loci in prostate cancer tissue, and our data provide the first suggestion of disrupted imprinting patterns in cancer for four imprinted genes (*DLK1*, *PLAGL1*, *SLC22A18*, and *TP73*). Although our results need to be further confirmed by larger studies, these findings suggest a more widespread dysregulation of genomic imprinting in prostate cancer than previously reported. Future investigations such as studying the biological significance of dysregulated imprinting genes are also warranted.

## Competing interests

The authors declare that they have no competing interests.

## Authors’ contributions

DIJ analyzed the data and prepared the manuscript. YM and AF were involved in data analysis. WKK and YZ designed the study and were involved in data analysis, interpretation, and manuscript preparation. All authors read and approved the final manuscript.

## Pre-publication history

The pre-publication history for this paper can be accessed here:

http://www.biomedcentral.com/1471-2490/13/37/prepub
